# Reproductive toxicity of seafood contaminants: Prospective comparisons of Swedish east and west coast fishermen's families

**DOI:** 10.1186/1476-069X-7-20

**Published:** 2008-05-28

**Authors:** Anna Axmon, Lars Rylander, Anna Rignell-Hydbom

**Affiliations:** 1 Division of Occupational and Environmental Medicine, Department of Laboratory Medicine, Lund University, University Hospital, SE-221 85 Lund, Sweden

## Abstract

Cohorts comprising fishermen's families on the east coast of Sweden have been found to have a high consumption of contaminated fish as well as high body burdens of persistent organochlorine pollutants (POPs). Their west coast correspondents are socio-economically similar, but with considerably lower POP exposure since the fish caught on the west coast is far less contaminated. The rationale for this was that the cohorts residing on the east coast of Sweden have been found to have a high consumption of contaminated fish as well as high body burdens of POPs, whereas their west coast correspondents are socio-economically similar, but with considerably lower POP exposure since the fish caught on the west coast is far less contaminated. Among the reproductive outcomes investigated are included both male and female parameters, as well as couple fertility and effects on the fetus. A range of exposure measures, including both questionnaire assessments of fish consumption and biomarkers, have been used.

The most consistent findings of the studies are those related to the fetus, where a decreased birth weight was found across all measures of exposure, which is in agreement with studies from other populations. Some markers for male reproduction function, i.e. sperm motility, sperm chromatin integrity, and Y:X chromosome ratio, were associated with POP exposure, whereas others, such as sperm concentration and semen volume, were not. With respect to couple fertility and female reproductive parameters, no support was given for associations with POP exposure. Although some associations may have been affected by beneficial effects of essential nutrients in seafood, the overall findings are meaningful in the context of reproductive toxicity and support the usefulness of the epidemiological design.

## Introduction

Concerns regarding health effects of persistent organochlorine pollutants (POPs) were first expressed in the 1960's when exposed animals were found to be affected, and exhibiting thinning eggshells, morphologic abnormalities, and impaired viability of offspring [1]. Since then, the topic has been thoroughly investigated in experimental animal studies as well as epidemiological animal and human studies. Several POPs are endocrine disruptors, *i.e*. chemicals with sex steroid mimicking effects which may cause developmental and reproductive abnormalities in humans and animals [2-4].

The term "persistent organochlorine pollutants" (POPs) is often used to include several compounds, such as polychlorinated biphenyls (PCBs), dioxins and organochlorine insecticides such as 1,1,1-trichloro-2,2-bis(p-chlorophenyl)ethane (DDT). Although they have had different applications in the industry, POPs have several traits in common: They are lipophilic and poorly metabolized, and they bioaccumulate. While they are not very soluble in water, they have spread widely through the atmosphere, and are found in parts of the world where they were never used [5]. Moreover, even after their use has been banned, POPs are still found in human and animal tissue and in body fluids [6-10].

The most common exposure route for the majority of individuals is through the consumption of contaminated foods. In common, for some of the most highly exposed populations, such as Inuits from Greenland and individuals from Faroe Islands, is that the main exposure source is from the seafood [9,11]. In Sweden, the major exposure source is consumption of fatty fish from the Baltic Sea, off the east coast of the country. As a consequence, plasma levels of several of the compounds were higher in men with a high intake of such fish than in those who consumed only moderate amounts, and the levels were higher in those who ate moderate amounts of fish than in those who ate none [12].

It has been found that both professional east coast fishermen and their wives have a higher intake of Baltic Sea fish than referents from the same area [13,14]. Similarly, on the west coast of Sweden, fishermen and their wives have a higher consumption of locally caught fish than referents from the same area. The average amount of locally caught fish consumed by fishermen and their wives on the east and west coast, respectively, has been found to be similar. However, the fish caught off the west coast of Sweden has for the past decades been much less contaminated than that caught from the Baltic Sea [15]. Thus, east coast fishermen have been found to have higher levels of POPs than both west coast fishermen and referents [13,16]. Consequently, the east coast fishermen and their wives constitute a suitable exposed group for investigating health effects of adult dietary exposure to POPs, whereas their counterparts on the west coast is an appropriate referent group. The socio-economic similarities between these cohorts in combination with a broad exposure range of POPs, gave very good opportunity to investigate the hypothesized association between POP exposure and reproductive health. Moreover, east coast fishermen's sisters have been found to be more likely to have grown up in a fishing village and/or fisherman's family [17], suggesting that they may be a fitting group when investigating health effects due to a high dietary exposure to POPs during childhood and adolescence, or even in utero. Therefore, the cohorts of fishermen, their wives and sisters, from the two coastal areas of Sweden (The Swedish Fishermen's Families Cohorts) have over the last two decades been used to study a variety of health effects.

### Study populations

In 1988, two cohorts of fishermen from the east (Baltic Sea) and west (Kattegatt and Skagerrak) coasts of Sweden were established [13,18]. Cohort affiliation was determined by the particular organization the fisherman was associated with. All in all, 2883 east coast fishermen and 8477 west coast fishermen were included in the cohorts. However, a subgroup of 96 east and 99 west coast fishermen also participated in a second study, in which both semen and blood samples were collected [16,19-21].

The fishermen cohorts were linked to the national Swedish Population Register and to registers at the local parish offices [22]. Thus, 2175 east and 7062 west coast women who were, or had been, married to a man in one of the original fishermen cohorts were identified. Cohort affiliation was determined by that of the fisherman to whom the woman was associated. Subgroups of these women have supplied blood samples [23] and information on time to pregnancy (TTP) [24]. Moreover, the entire cohorts of fishermen's wives were linked to the Swedish Medical Birth Registry (MBR) [14], thus identifying 1501 infants born to the east coast women during 1973 through 1991, with the corresponding number for the west coast being 3553. The children were assigned the same cohort affiliation as their mothers.

In 1996, the cohorts of fishermen were linked to the National Swedish Population Register in order to identify sisters (at least one parent in common) to these men [17]. Women who were included in the fishermen's wives cohort were excluded. The cohort affiliation was determined by that of the fisherman to whom the woman was associated. The cohorts of sisters were then linked to the MBR, and 1719 east and 1537 west coast infants were identified. Also among the fishermen's sisters a study regarding TTP was performed, including 1241 east and 2023 west coast women [25]. Of these, 165 women also provided blood samples [26].

### Measures of exposure

In the studies performed on the Swedish Fishermen's Families Cohorts, several measures of exposure have been used (c.f. Table 1). In those studies which rely on register data and/or questionnaires and telephone interviews, the main measure of exposure has been *cohort affiliation*, using the east coast cohorts as exposed cohorts and the west coast cohorts as referent cohorts. Moreover, within the east coast cohorts, *growing up in a fishing village and/or fisherman's family *has been used as a proxy measure of exposure during childhood and adolescence. In one of the earlier studies on the fishermen's wives, it was found that recall of fish consumption was poor [23]. Thus, in subsequent studies, *current fish consumption *was used as a proxy measure of exposure during the time of interest for each specific outcome (e.g. pregnancy attempt or child birth).

**Table 1:** Reproductive outcomes investigated in the Swedish Fishermen's Families Cohorts.

		Exposure				
Unit of observation	Outcome^1^	Cohort affiliation	Childhood exposure^2^	Fish consumption	CB-153^3^	p,p'-DDE^4^
Man	Sperm motility	-	-	-	↓ [16]	↓ [16]
	Sperm concentration	-	-	-	0 [16]	0 [16]
	Semen volume	-	-	-	0 [16]	0 [16]
	Total sperm count	-	-	-	0 [16]	0 [16]
	Sperm chromatin integrity	-	-	-	↓ [20]	(↓) [20]
	Y:X chromosome ratio	-	-	-	↓ [21]	↓ [21]
	Seminal levels of markers of epididymal and accessory sex gland function	-	-	-	0 [19]	0 [19]
	Hormone parameters	-	-	-	0 [16]	0 [16]
Woman	Age at menarche	0 [38]	↓ [38]	-	-	-
	Menstrual cycle length	↓ [91]	0 [91]	(↓) [91]	0 [91]	-
	Miscarriages and stillbirths	NC [17, 25, 40]	0 [25, 40]	0 [25]	NC [26, 40]	-
Couple	Time to pregnancy	NC [24, 25]	0 [24, 25]	0 [24, 25]	0 [26, 33]	-
Infant/child	Birth weight	↓ [14, 17]	↓ [41]	↓ [41]	↓ [31]	-
	Birth length	0 [14, 17]	-	-	-	-
	Head circumference	↓ [14, 17]	-	-	-	-
	Gender ratio	NC [14, 17]	-	-	-	-
	Malformations	0 [17, 43]	-	-	-	-

^1 ^Arrows indicate the effect associated with each exposure, with parenthesis for those effects not found to be of a noteworthy size but not statistically significant. A "0" means that no effect was found, and "NC" signifies non conclusive results.

^2 ^Growing up in a fisherman's family and/or fishing village

^3 ^2,2',4,4',5,5'-hexachlorobiphenyl

^4 ^1,1-dichloro-2,2-bis (*p*-chlorophenyl)-ethylene

With respect to the majority of the reproductive outcomes investigated, *biomarkers *have also been used as measures of exposure. The main biomarker has been 2,2',4,4',5,5'-hexachlorobiphenyl (CB-153). This congener has been found to correlate strongly with the total PCB concentration, as well as the total PCB-derived TEQ and the total POP-derived TEQ, in humans [27-30], and has therefore been considered not only a biomarker of PCBs, but of overall POPs. Moreover, the major DDT metabolite 1,1-dichloro-2,2-bis (*p*-chlorophenyl)-ethylene (p,p'-DDE) which has anti-androgenic effect and is found in high levels in serum, has also been used. In the studies, both biomarkers were used in their lipid adjusted form, i.e., the wet weight concentrations were standardized using the sum of serum concentrations of triglycerides, cholesterol and phospholipids [27].

Since many of the studies on the Swedish Fishermen's Families Cohort have been of a retrospective nature, a drawback is that the biomarkers describe current, rather than past, exposure. Therefore, a model to estimate past exposure was derived [31]. This model assumed a constant consumption of fatty fish, and a constant background exposure. However, the model took into consideration the decreasing levels of POPs in fatty fish, the reduction of body burden due to lactation, and the half-life of the compound in humans. The model was validated for CB-153 in women by comparing current concentrations of CB-153 to concentrations obtained from bio-banked samples from a rubella screening performed years earlier. The model has since then been validated for male CB-153, as well as for both male and female p,p'-DDE [32]. For CB-153, the correlations between biobanked (i.e. true) and estimated concentrations were 0.96 for men and 0.97 for women. When trichotomizing the female CB-153 concentrations, the kappa agreement between biobanked and estimated concentrations was 0.47 [33]. For p,p'-DDE the correlation between biobanked and estimated concentrations was 0.78 for both men and women [32].

The median plasma concentration of CB-153 among the fishermen' wives from the Swedish east coast was 160 ng/g lipid (range 16–780) and the median serum concentration among the sisters to the fishermen from the Swedish east coast was 115 ng/g lipid (range 27–560). With respect to the estimated past exposures, this varied from study to study depending on the time for which the concentrations were estimated, and which parameters were used in the model. In those studies where estimated past exposure was related to any outcome, the estimated past concentrations was between 36% and 124% higher than the measured. The median serum concentrations among the fishermen were 193 ng/g lipid (range 40–1460) for CB-153 and 240 ng/g lipid (range 40–2250) for p,p'-DDE.

### Male reproduction parameters

In the studies concerning male reproductive parameters, serum concentrations of CB-153 and p,p'-DDE were analyzed both as continuous measures (untransformed or log transformed) and categorized into five equally sized groups: For CB-153 the cut-points were 112, 167, 232, and 328 ng/g lipid, and for p,p'-DDE 136, 191, 273, and 471 ng/g lipid [16,19-21]. The correlation between CB-153 and p,p'-DDE for exposure was strong (r = 0.78). Thus, the two biomarkers were never used simultaneously in any model.

A negative association, although not statistically significant when age was taken into account, between serum concentrations of CB-153 and the proportion of motile sperm was found [16]. Similar results were found for p,p'-DDE. No consistent associations were found between any of the two biomarkers and semen volume, sperm concentration, total sperm count or sperm morphology [16,34].

Sperm Chromatin Structure Assay (SCSA) was used to detect DNA fragmentation (small breaks in the sperm chromosome) [35,36]. The DNA fragmentation index (%DFI) was used to express the percentage of sperms showing DNA fragmentation [20]. Men in the lowest CB-153 quintile had lower %DFI than the men with higher CB-153 concentrations [20]. The pattern was similar for p,p'-DDE. With respect to continuous measures of CB-153 and p,p'-DDE there were no consistent association with %DFI.

Two color fluorescence in situ hybridization (FISH) was used to assess sperm chromosome Y:X ratio in an ejaculate [21]. Both CB-153 and p,p'-DDE were found to associate with an increased Y chromosome fraction [21,37]. When comparing the lowest exposed quintile to the highest, the effect was more marked for p,p'-DDE than for CB-153 [21]. Fructose, zinc, prostate specific antigen (PSA), and neutral α-glucosidase (NAG) were analyzed as markers for the function of the epididymis, prostate and seminal vesicles [19]. Among these outcomes, the only significant association was found between CB-153 and total PSA [19].

When age was taken into account, no significant associations were observed between the POP biomarkers and serum hormone levels (FSH, LH, estradiol, testosterone, SHBG, inhibin B, and the ratio testosterone/SHBG, respectively) [16].

### Female reproduction parameters

East coast fishermen's wives and fishermen's sisters, who had grown up in a fishing village and/or fisherman's family tended to be somewhat older at menarche than referents from the same coastal area [38]. However, no differences were found between these women and the women from the west coast.

When adjusting for smoking, east coast fishermen's wives and sisters had somewhat shorter menstrual cycles than the west coast women. Moreover, in the east coast cohorts, women with a high consumption of fatty fish had shorter menstrual cycles than those with no or low fish consumption. However, there were no difference between women who had and had not grown up in a fishing village and/or fisherman's family, neither was there any effect of serum/plasma concentrations of CB-153.

There were no increased risk for miscarriage and stillbirths among the east coast fishermen's wives and fishermen's sisters, as compared to the corresponding cohorts from the west coast [25,39,40].

### Couple fertility

A decreased fertility (i.e. prolonged TTP) was found for the east coast fishermen's wives as compared to the west coast fishermen's wives (Cox regression; Success Rate Ratio [SuRR] 0.86; 95% CI: 0.75, 0.99) [24]. The effect was most apparent among women who smoked more than 10 cigarettes per day, i.e. comparing east and west coast smokers (SuRR 0.68; 95% CI: 0.51, 0.91). Among the fishermen's sisters, no effect was found for cohort affiliation (logistic regression; Fecundability Odds Ratio [FOR] 0.99; 95% CI: 0.87, 1.14) [25]. The effect among smokers was not apparent in the group of fishermen's sisters. Among the east coast women, growing up in fishing village and/or fisherman's family did not suggest any negative effect on TTP, neither did a high consumption of fatty fish [24,25,39] or serum/plasma concentrations of CB-153 [26,33,39].

### Effects on the fetus

Among the east coast fishermen's wives, the median birth weight was found to be 3530 g, which was lower than the corresponding figure for the west coast fishermen's wives (3610 g; p < 0.001) [14]. The absolute birth weight difference was slightly lower among the fishermen's sisters (3500 g for the east coast women and 3560 g for the west coast women), although the statistical testing still found it significant (p < 0.001) [17].

In a separate case-control analysis, a high total current intake of fish from the Baltic Sea indicated an increased risk of having an infant with low birth weight (OR = 1.9; 95% CI: 0.9, 3.9), although no dose-response relation was found [41]. Furthermore, when analyzing estimated intake of fish during the time period of interest (i.e. when the infant was born), the effects were diminished. However, childhood exposure (measured as growing up in a fishing village) carried an increased risk of giving birth to a low birth weight infant (OR = 2.1; 95% CI: 1.0, 4.3). The case mothers had higher median concentrations of CB-153 than the control mothers (190 ng/g lipid versus 160 ng/g lipid) [31].

There were no differences in birth length between the cohorts [14,17]. Among the fishermen's wives as well as the fishermen's sisters, the distribution of head circumference of the east coast infants was shifted downwards compared to the west coast infants (p < 0.001 for both groups of women). An increased risk of being small for gestational age (SGA) [42] was found among infants born to the east coast fishermen's wives and sisters (OR = 1.5; 95% CI: 1.2, 2.0) [17]. Overall, there were no clear deviating patterns regarding gender ratio [14,17]. No specific malformation was overrepresented in the east coast cohorts [17,43].

### Discussion

The Swedish Fishermen's Families Cohorts consist of a large number of men and women of varying ages. The east coast cohorts comprises individuals who have been exposed to POPs at different times in their lives; those who are born and grown up in a fisherman's family and/or a fishing village are likely to have been exposed to POPs in utero and during their childhood, whereas others may have had high exposure only during their adult life. These assumptions are supported by that the men and women in the east coast cohorts have been found to have higher average levels of POPs than men and women from the general Swedish population [27,44,45], and those who have grown up in a fishing village and/or fishermen's family have been found to have especially high POP levels [45]. Thus, a major strength with the Swedish Fishermen's Families studies is the use of a large and relatively highly exposed study population.

The men and women in the east coast Swedish Fishermen's Families Cohorts have been found to eat more fish than individuals from the general Swedish population [14,17,22]. Since fish have constituents, e.g. long-chain n-3 fatty acids, minerals and vitamins, which may have positive effects on reproductive health [46-48], the general population may not be a suitable reference group in studies of health effects of dietary exposure to POPs. However, the waters off the west coast of Sweden are considerably less contaminated than the Baltic Sea [15]. The west coast Swedish Fishermen's Families Cohorts have been found to eat fatty fish at a rate comparable to that of the east coast cohorts [14,22], but to have considerably lower levels of POPs [12]. Geographically, Sweden is a narrow country, only approximately 500 km at the widest location. Of the 9.1 million population, only 17% have non-Swedish origin [49]. The largest city, Stockholm, is inhabited by slightly more than 1 million individuals, making it a rather small city in an international perspective. All these factors taken together suggest that the Swedish population is a comparably homogenous one. With respect to the Swedish Fishermen's Families, the east and west coast cohorts, although residing at different coast lines, are geographically close. Moreover, although the east coast fishermen's wives tend to smoke more than those in the west coast cohorts [22,40], the west coast cohorts have been found to be similar to the east coast cohorts with respect to other socioeconomic factors, such as alcohol consumption [14,17,22] and educational level [17,40]. Hence, another major strength with the Swedish Fishermen's Families studies is the use of large and socio-economically similar cohorts as referent groups.

In several of the studies performed on the Swedish Fishermen's Families Cohorts, biomarkers for exposure to POPs have been used. Most frequently, CB-153 has been considered as a biomarker for a person's total body burden of POP. The rationale for this is that this PCB congener has been found to correlate strongly with the total PCB concentration in plasma [27], serum [28], whole venous blood [50], and cord blood [50], as well as with the TEQ from total PCB [27,30] and total POP-derived TEQ [6]. Other proxy measures of exposure used in the Swedish Fishermen's Families Cohorts, i.e. cohort affiliation, fish consumption and grown up in a fisherman's family and/or fishing village, have been found to be strongly associated with the concentrations of CB-153 [12,27,45]. Thus, a further strength with the Swedish Fishermen's Families Cohorts studies is the use of biomarkers of exposure. However, the studies are in general retrospective or cross-sectional and the proper exposure window might not always be reflected in an optimal way. For instance, in the study which investigated the association between POP exposure and low birth weight, we had to estimate the concentrations of CB-153 during year of childbirth based on back-calculation models [31]. This might led to a negative impact on precision, as well as accuracy, of the estimated CB-153 concentrations. As stated by Grandjean et al [51], it is probably not only the dose that makes the poison, of equally importance might be the timing of exposure. Accordingly, future studies should force to collect exposure information that in a better way reflect the most relevant exposure window.

When they enter the human body, POPs are stored in the adipose tissue. Thus, to obtain a valid measure of the body burden of POP, a biopsy should be performed and fatty tissue should be analyzed. This is, however, a procedure which is more costly and painful than collecting a blood sample. Nevertheless, it may be assumed that the concentration of POP in serum lipids is in equilibrium with that in adipose tissue. Kahn et al [52] found a very high correlation (r = 0.89) between 2,3,7,8 TCDD concentration in fat biopsy and the lipid adjusted serum concentration. Thus, the body burden of POP may be estimated by calculating the lipid standardized serum concentrations. However, Schisterman et al [53] have recently shown that this method is highly prone to bias, causing an underestimation of the true effect. Nevertheless, the correlations between wet weight and lipid adjusted concentrations of POPs in the Swedish Fishermen's Families Cohorts are high [27,45]. Moreover, the results obtained when using the biomarkers are in agreement with those obtained using the proxy measures of exposure. Thus, a possible bias introduces by the lipid standardization should pose, at most, a minor problem.

A variety of outcomes have been investigated in the Swedish Fishermen's Families Cohort. The ones chosen have been such that they are believed to cover all aspects of human reproduction, i.e. parameters concerning the man, the woman, the couple and the fetus. By using this multitude of outcomes, we have been able to establish where POPs may do most harm to human reproduction, i.e. to the fetus. However, the retrospective design may pose a problem for some outcomes, where recall was long and a possible misclassification may have been introduced. The most apparent example of this is menarche. For other outcomes, mainly those related to the fetus, data has been collected from registries and the quality should therefore be higher. Also, the studies concerning male reproductive factors were not affected by any recall bias.

The most consistent results from the Swedish Fishermen's Families Cohorts are those on infant parameters, and especially birth weight and fetal growth. Irrespective of which measure of exposure was used, children who are exposed to POPs in utero have an increased risk of weighing less at birth, also after having taken the gestational length into account (SGA). Similar findings have been observed among fish eaters from the Lake Michigan area in the US who had PCB levels comparable to the ones among the Swedish fishermen's wives [54]. Also in the general population in the Netherlands with relatively low POP exposures, in utero levels of PCB were negatively associated with birth weight [55]. There are, however, also epidemiological examples showing no, or even positive, associations between POP exposure and birth weight [56-58]. The overall picture regarding the hypothesized association between POP exposure and birth weight is, accordingly, still far from conclusive.

Environmental chemicals acting as sex hormone agonists or antagonists could have an adverse effect on male sex hormones which in turn might decrease the male reproductive function [4,59]. In our study sperm motility decreased with increasing serum levels of POP. This finding is consistent with four other cross-sectional studies carried out during recent years [60-63]. Sperm DNA integrity is essential for the accurate transmission of genetic information and sperm chromatin abnormalities or DNA damage may result in impaired male fertility [64]. Among Swedish fishermen we found that increasing POP exposure increased the level of sperm DNA strand breaks. However, in a study comprising Inuits, with higher serum levels of POP, the levels of sperm DNA strandbreaks were low. This inconsistent result might be explained by differences in the genetic background and lifestyle habits, which still needs to be elucidated. The mechanism behind changes in Y:X ratio is unknown. However one hypothesis could be a loss of X chromosome due to an effect on formation of micronuclei during the meiosis, by *e.g*. POPs. We found effects of POP biomarkers on the proportion of Y- and X-bearing sperm. However, such effects were not seen in three other study populations [65]. The inconsistent results might be explained by differences in POP exposure profile and dose due to variation in lifestyle and diet. Regarding sperm count and sperm morphology none of these outcomes were related to POP exposure.

The results concerning female reproductive parameters are ambiguous, and in some cases they suggest a protective, rather than hazardous, effect of fish consumption. Taking other studies on the topic into account, no obvious risk pattern emerges for most of the outcomes. Although results from a cross-sectional study on Akwesasne Mohawk girls suggested that high concentrations of blood PCB lead to a higher probability of early menarche [66], retrospective studies performed by Vasiliu et al [67] and in the Swedish Fishermen's Families Cohort studies found no such effect. Moreover, whereas exposure to POPs was associated with a shortened menstrual cycle length in the retrospective studies performed in the Swedish Fishermen's Families Cohorts and the New York State Angler Cohort [68], another retrospective study found the opposite relation between exposure and outcome [69], whereas a prospective study using daily urine sampling found no relation at all [70]. With respect to miscarriages and stillbirths, the results available are more homogenous than those for the other outcomes. In the Swedish Fishermen's Families Cohorts, none of the analyses produced a statistically significant hazard associated with POP exposure. However, in some of the subgroup analyses, POP exposure was suggested to have a protective effect. This is in agreement with other studies, were no effect [71-73], or a protective effect [74], has been found.

East coast fishermen's wives were found to have longer TTPs than west coast fishermen's wives. However, these results were not replicated among the fishermen's sisters, neither did any of the exposure measures within the east coast cohorts suggest any effect of POP exposure. In the New York State Angler Cohort, several studies have been performed regarding parental POP exposure and fertility. The majority of these results show no effect on TTP, delayed conception, or infertility of maternal [75,76] or paternal [77,78] exposure, although one study did suggest reduced fecundability among women with high consumption of fatty fish from the Great Lakes [78]. In the INUENDO study, results suggested that high exposure to POPs were associated with reduced fertility only among Inuits from Greenland, and not among couples, i.e. maternal and paternal exposure, from Poland, Ukraine or Sweden [79].

When evaluating the results from the Swedish Fishermen's Families Cohorts studies, it is important to keep in mind that the main exposure source for POP, namely fatty fish from the Baltic Sea, is also an exposure route for other constituents of fish. These other constituents include both other pollutants, such as methylmercury, and natural components, such as marine n-3 fatty acids, minerals and vitamins. In the Swedish Fishermen's Families Cohorts, the concentration of CB-153 has been found to be correlated (r_S _= 0.51) to that of methylmercury [80]. However, where associations were found between on one hand CB-153 and p,p'-DDE and on the other hand several male reproductive parameters, no consistent relationships were found between methylmercury and the same male parameters.

With respect to the natural components of fatty fish, marine n-3 fatty acids have been found to be associated with prolonged gestation in randomized trials [81-85]. Moreover, positive associations between seafood consumption and reproductive outcomes, such as birth weight and gestational length, have been seen in observational studies [47,48,86-90]. Thus, there is a possibility that the effect of the potentially hazardous constituents in Baltic Sea fatty fish could be outweighed by their beneficial counterparts, which would explain the positive results found for some outcomes.

## Conclusion

The major strengths of the studies were that the study populations included socio-economic similar cohorts, which enabled good opportunities to control for confounding, and the cohorts included the necessary exposure contrasts, which made it possible to investigate the hypothesized association between POP exposure and reproductive health.

The main finding from the studies performed on the Swedish Fishermen's Families Cohorts is that although there may be minor effects on both the male and female reproduction system, the major effects are those concerning the growth of the fetus. This observation was strengthened by the fact that the negative effect on birth weight was observed for all exposure measures used. In addition, corresponding effects have been observed in other populations with a similar exposure source, i.e. intake of contaminated fish.

Regarding male and female reproductive data, the Swedish data together with other epidemiological studies, provide no conclusive results. Although some associations may have been affected by beneficial effects of essential nutrients in seafood, the overall findings are meaningful in the context of reproductive toxicity and support the usefulness of the epidemiological design.

## Competing interests

The authors declare that they have no competing interests.

## Authors' contributions

AA initiated the project. All authors participated in drafting the manuscript, and have approved of the final version.
